# Chloroplast genome variation and phylogenetic relationships of *Atractylodes* species

**DOI:** 10.1186/s12864-021-07394-8

**Published:** 2021-02-04

**Authors:** Yiheng Wang, Sheng Wang, Yanlei Liu, Qingjun Yuan, Jiahui Sun, Lanping Guo

**Affiliations:** 1 National Resource Center for Chinese Materia Medica, China Academy of Chinese Medical Sciences, Beijing, 100700 China; 2 State Key Laboratory of Systematic and Evolutionary Botany, Institute of Botany, Chinese Academy of Sciences, Beijing, 100093 China

**Keywords:** Traditional herbal medicine, Chloroplast markers, Simple sequence repeat, Indel, Interspecific relationships

## Abstract

**Background:**

*Atractylodes* DC is the basic original plant of the widely used herbal medicines “Baizhu” and “Cangzhu” and an endemic genus in East Asia. Species within the genus have minor morphological differences, and the universal DNA barcodes cannot clearly distinguish the systemic relationship or identify the species of the genus. In order to solve these question, we sequenced the chloroplast genomes of all species of *Atractylodes* using high-throughput sequencing.

**Results:**

The results indicate that the chloroplast genome of *Atractylodes* has a typical quadripartite structure and ranges from 152,294 bp (*A. carlinoides*) to 153,261 bp (*A. macrocephala*) in size. The genome of all species contains 113 genes, including 79 protein-coding genes, 30 transfer RNA genes and four ribosomal RNA genes. Four hotspots, *rpl22*-*rps19*-*rpl2*, *psbM*-*trnD*, *trnR*-*trnT*^*(GGU)*^, and *trnT*^*(UGU)*^-*trnL*, and a total of 42–47 simple sequence repeats (SSR) were identified as the most promising potentially variable makers for species delimitation and population genetic studies. Phylogenetic analyses of the whole chloroplast genomes indicate that *Atractylodes* is a clade within the tribe *Cynareae*; *Atractylodes* species form a monophyly that clearly reflects the relationship within the genus.

**Conclusions:**

Our study included investigations of the sequences and structural genomic variations, phylogenetics and mutation dynamics of *Atractylodes* chloroplast genomes and will facilitate future studies in population genetics, taxonomy and species identification.

**Supplementary Information:**

The online version contains supplementary material available at 10.1186/s12864-021-07394-8.

## Background

Chloroplasts are multifunctional organelles with independent genetic material, which are commonly found in terrestrial plants, algae and a few protozoa. There are multiple configurations of the chloroplast genome in the cell; the most common structure is double-stranded circular configuration including a small single copy region (SSC) and a large single copy region (LSC). These two regions are separated by a pair of inverted repeat regions (IRa, IRb) to form a typical quadripartite structure. The genome size ranges from 120 to 160 kb [1]. Compared with the mitochondrial or nuclear genome, the plant chloroplast genome has a higher conservation in terms of structure, gene number and gene composition. The evolution rate is relatively moderate and is between the nuclear and mitochondrial genome [2]. Due to the lack of recombination, small genome size and high copy number per cell [3, 4], complete chloroplast genome sequences have been extensively used in phylogenetics analysis and species identification [5, 6]. The results showed that the chloroplast genome contains additional information to improve phylogenetic analysis [7–11]. Comparative chloroplast genome sequences provide an opportunity to discover the sequence variation and identify mutation hotspot regions, while also detecting the gene loss and duplication events. Mutation hotspot regions and single sequence repeats (SSRs) obtained from the chloroplast genome sequences can be effective molecular markers for species identification and population genetics [12].

*Atractylodes* is a small East Asian endemic genus of the Asteraceae family with 6 species and is distributed in China, Japan, and the Korean Peninsula. Traditional Chinese herbal medicines “Baizhu” and “Cangzhu” originate from *Atractylodes* [13]. It is the traditional medicine for treatment of gastroduodenal diseases. All species of the genus have been used as an herbal medicine except *A. carlinoides*. The “Pharmacopoeia of the People’s Republic of China” states that “Cangzhu” is the dried rhizome of *A. lancea* and “Baizhu” is the dry rhizome of *A. macrocephala*. However, traditional medicine in Japan considers *A. lancea*, *A. coreana* and *A. chinensis* “Cangzhu” and *A. japonica* and *A. macrocephala* “Baizhu” [14]. Similar medicinal effects and mixed use reflect the complexity of the systematic relationship of the original plant. Indeed, the genus *Atractylodes* was identified as early as 1838; however, the relationship between and within the genus has never ceased to be controversial.

**Fig. 1:**
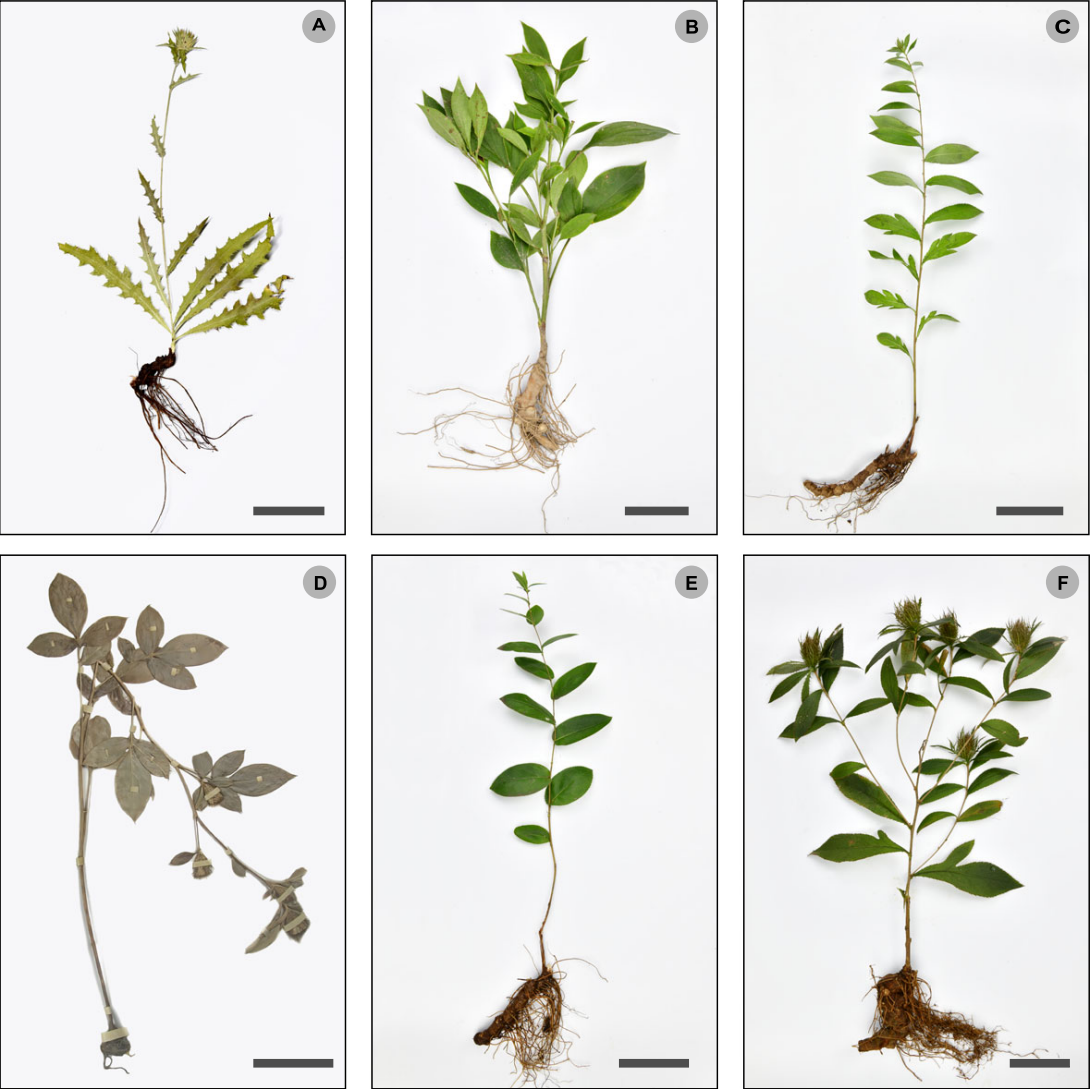
Comparison of vegetal morphologies among Atractylodes species. Scale bars are 5 cm. **A** *A. carlinoides*, **B** *A. macrocephala*, **C** *A. lancea*, **D** *A. japonica*, **E** *A. coreana*, **F** *A. chinensis*

The morphological variation in this genus is relatively large and the relationships are difficult to determine by traditional identification. *A. carlinoides* has pinnatifid, rosulate basal leaves, whereas *A. macrocephala* has branched stem from base, which easy to distinguish from other species (Fig. 1). But the other four species are difficult to distinguish from each other morphologically, especially when the plants are young and have unbranched stems and undivided leaves.

Several studies have used several chloroplast markers, such as *atpB-rbcL trnK*, *trnL-F,* and/or nuclear ITS, to determine the relationship of the genus [15–17]. However, the phylogenetic relationships within *Atractylodes* have been poorly defined because of limited number of DNA sequences and low number of the variation markers. In this study, we sequenced the chloroplast genome of all six *Atractylodes* species. The objectives of this study were (1) to compare the chloroplast genome of *Atractylodes* to understand the evolution of the genome structure, (2) to determine the highly variable regions for species identification, and (3) to clarify the phylogenetic relationship of *Atractylodes*.

## Results

### Chloroplast genome sequencing and features of *Atractylodes* species

Six *Atractylodes* species were used to obtain 10,016,902 - 44,594,826 raw reads with the average coverage of 67X - 1431X (Table 1). Six complete chloroplast genome sequences were deposited in GenBank with accession numbers MT834519 to MT834524. The total chloroplast genome size ranged from 152,294 bp (*A. carlinoides*) to 153,261 bp (*A. macrocephala*). The *Atractylodes* chloroplast genome has a typical quadripartite structure and includes a pair of IR regions (25,132 bp - 25,153 bp), LSC regions (83,359 bp - 84,281 bp) and SSC regions (18,634 bp - 18,707 bp). The average GC content is 37.7% in the total chloroplast genome, 43.2% in IR, 35.8–35.9% in LSC, and 31.4–31.6% in SSC; there are almost no differences between the six *Atractylodes* chloroplast genomes.

**Table 1:** The basic chloroplast genome information of six *Atractylodes* species

Characteristics	***A. chinensis***	***A. coreana***	***A. lancea***	***A. macrocephala***	***A. japonica***	***A. carlinoides***
Raw data no.	10,016,902	38,042,502	42,933,804	44,594,826	12,772,648	15,350,264
Mapped read no.	373,164	262,561	1,142,990	1,462,514	68,040	116,137
Percent of chloroplast genome reads(%)	3.73	0.69	2.66	3.28	0.53	0.76
Chloroplast genome coverage(X)	365	257	1119	1431	67	114
Total size(bp)	153,177	153,201	153,181	153,261	153,198	152,294
LSC length(bp)	84,241	84,198	84,255	84,281	84,254	83,359
IR length(bp)	25,147	25,148	25,146	25,153	25,140	25,132
SSC length(bp)	18,642	18,707	18,634	18,674	18,664	18,671
Total genes	113	113	113	113	113	113
Protein coding genes	79	79	79	79	79	79
tRNA genes	30	30	30	30	30	30
rRNA genes	4	4	4	4	4	4
Overall GC content(%)	37.70%	37.70%	37.70%	37.70%	37.70%	37.70%
GC content in LSC(%)	35.80%	35.80%	35.80%	35.80%	35.80%	35.90%
GC content in IR(%)	43.20%	43.20%	43.20%	43.20%	43.20%	43.20%
GC content in SSC(%)	31.50%	31.50%	31.50%	31.60%	31.60%	31.36%
Accession number	MT834519	MT834521	MT834522	MT834520	MT834523	MT834524

**Fig. 2:**
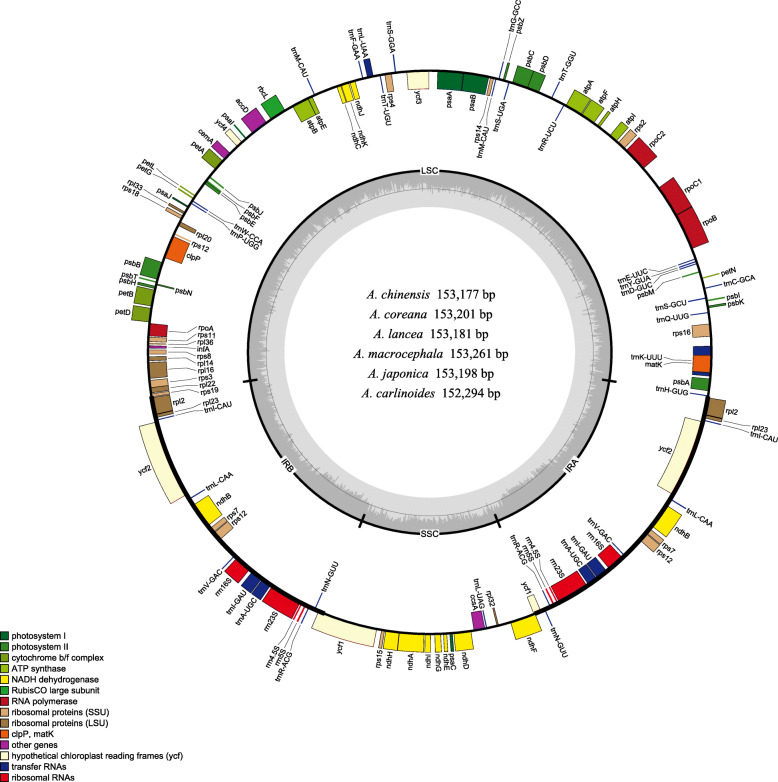
Gene maps of the chloroplast genomes of *Atractylodes*. Genes on the inside of the large circle are transcribed clockwise and those on the outside are transcribed counter clockwise. The genes are color-coded based on their functions. The dashed area represents the GC composition of the chloroplast genome

The chloroplast genome of *Atractylodes* has 113 genes, including 79 protein-coding genes, 30 transfer RNA genes and four ribosomal RNA genes (Fig. 2, Table 2). Six protein-coding genes (*ndhB*, *rpl23*, *rps7*, *rps12*, *ycf2*, and *rpl2*), seven tRNA genes (*trnI-CAU*, *trnL-CAA*, *trnV-GAC*, *trnI-GAU*, *trnA-UGC*, *trnR-ACG* and *trnN-GUU*) and all four rRNA genes are duplicated in the IR regions. Fourteen genes (*atpF*, *rpoC1*, *ndhB*, *petB*, *rpl2*, *ndhA*, *rps12*, *rps16*, *trnA-UGC*, *trnI-GAU*, *trnK-UUU*, *trnL-UAA*, *trnG-GCC* and *trnV-UAC*) contain a single intron and two genes (*clpP* and *ycf3*) have two introns. The *rps12* gene is a trans-spliced gene with 5′-end located in the LSC region and the 3′ end located in the IR region. The gene *trnK*-*UUU* has the largest intron, which contains the *matK* gene.

**Table 2:** The basic chloroplast genome information of six Atractylodes species

Category for genes	Group of genes	Name of genes
Photosynthesis related genes	Rubisco	*rbcL*
	PhotosystemI	*psaA,psaB,psaC,psaI,psaJ*
	Assembly/stability of photosystemI	*ycf3,ycf4
	PhotosystemII	*psbA,psbB,psbC,psbD,psbE,psbF,psbH,psbI,psbJ,psbK,psbL,psbM,psbN,psbT,psbZ*
	ATP synthase	atpA, atpB, atpE, *atpF, atpH, atpI
	cytochrome b/f compelx	petA, *petB, *petD, petG, petL, petN
	cytochrome c synthesis	*ccsA*
	NADPH dehydrogenase	*ndhA, *ndhB, ndhC, ndhD, ndhE, ndhF,ndhG, ndhH, ndhI, ndhJ, ndhK
Transcription and translation related genes	transcription	rpoA, rpoB, *rpoC1, rpoC2
	ribosomal proteins	rps2, rps3, rps4, rps7, rps8, rps11, *rps12, rps14,rps15, *rps16, rps18, rps19,*rpl2, rpl14, *rpl16, rpl20, rpl22, rpl23, rpl32, rpl33,rpl36
	translation initiation factor	*infA*
RNA genes	ribosomal RNA	*rrn5, rrn4.5, rrn16, rrn23*
	transfer RNA	*trnA-UGC, trnC-GCA, trnD-GUC, trnE-UUC, trnF-GAA, *trnG-UCC, trnG-GCC, trnH-GUG, trnI-CAU, *trnI-GAU,*trnK-UUU, trnL-CAA, *trnL-UAA, trnL-UAG, trnfM-CAUI,trnM-CAU, trnN-GUU, trnP-UGG, trnQ-UUG,trnR-ACG, trnR-UCU, trnS-GCU, trnS-GGA, trnS-UGA, trnT-GGU,trnT-UGU, trnV-GAC, *trnV-UAC, trnW-CCA, trnY-GUA
Other genes	RNA processing	*matK*
	carbon metabolism	*cemA*
	fatty acid synthesis	*accD*
	proteolysis	*clpP
Genes of unknown function	conserved reading frames	*ycf1, ycf2*

Intron-containing genes are marked by asterisks (*)

### Indels

**Fig. 3:**
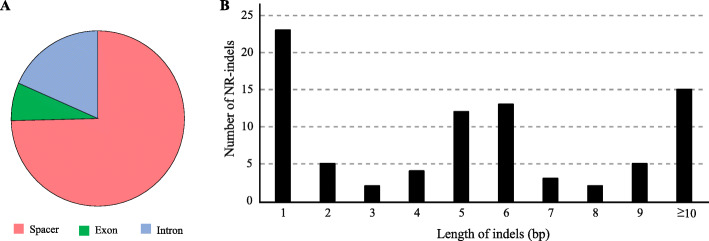
Analyses of indels in the *Atractylodes* chloroplast genomes. (A) Frequency of indel types and locations. (B) Number and size of non-SSR-related indels in the six *Atractylodes* chloroplast genomes

There are 114 indels in six *Atractylodes* chloroplast genomes, including 30 SSR-related indels (26.3%) and 84 non-SSR-related indels (73.7%); 74.6% indels are present in 42 intergenic space regions, 7.0% indels are located in exons, and 18.4% are present in the introns (Fig. 3a, Table S1). The *trnT-trnL* gene contains six indels; the *trnE*-*rpoB*, *ndhC*-*trnM* and *ycf1* genes contain 5 indels followed by the *rpl32*-*ndhF* and *trnL*-*rpl32* genes with 4 indels.

All SSR-related indels are single nucleotide size except an indel located in the ndhB-trnL region, which is 6 bp in size. The majority of the SSR-related indels are related to the A/T type SSRs (28 times). All SSR-related indels are located in the non-coding regions.

The size of the non-SSR-related indels ranges from 1 to 971 bp, with one bp indels being the most common (Fig. 3b). The largest indel (971 bp) in the spacer of *ndhC*-*trnM* is a deletion in *A. carlinoides*. The second largest indel is in the exon of *ycf1* with 30 bp size and is a deletion in *A. lancea* and an insertion in *A. coreana*. The majority of the NR-indels are located in the noncoding regions (91.67%), including 73.81% in the intergenic spaces and 17.86% in introns.

### SSRs

**Fig. 4:**
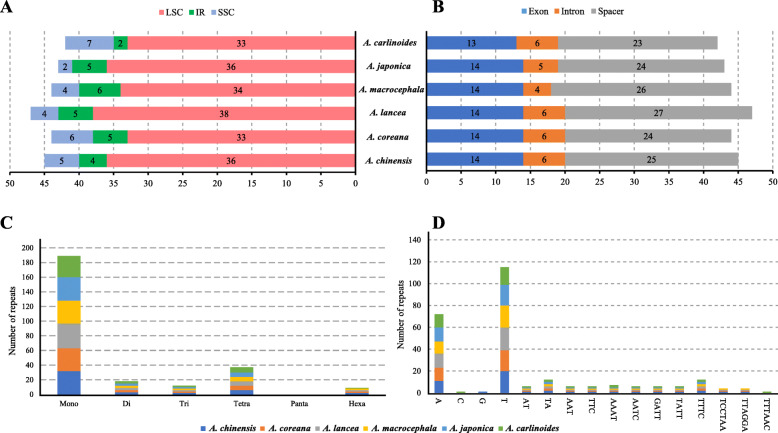
The type and distribution of SSRs in the six *Atractylodes* chloroplast genomes. (A) Frequency of SSR occurrence in the LSC, SSC, and IR regions. (B) Proportion of SSR distribution in various species (C) Number of SSR repeat types. (D) Number of identified SSR motifs in different repeat class types

A total of 265 SSRs were detected in the chloroplast genomes of six *Atractylodes* species by the GMATA analysis. The number of SSRs ranges from 42 (*A. carlinoides*) to 47 (*A. lancea*). SSR events are distributed randomly in the chloroplast genome. There are 210 SSRs in LSC, 28 in SSC, and 27 in the IR region (149 in spacers, 33 in introns and 83 in exons). With regard to individual genomes, the majority of SSRs were detected in LSC (ranging from 75.0% in *A. lancea* to 83.7% in *A. japonica*) and in spacers (ranging from 54.5% in *A. lancea* to 59.1% in *A. macrocephala*) (Fig. 3a). The most common SSRs are mononucleotides, which account for 71%, followed by tetranucleotides accounting for 14%, and dinucleotide SSRs accounting for 7% (Fig. 4b). Nearly all mononucleotide SSRs (99%) are composed of A and T in all six species. The dinucleotide repeats of TA and the tetranucleotide repeats of TTTC are the second most common SSRs (Fig. 4c).

### Sequence divergence and hotspots

**Fig. 5:**
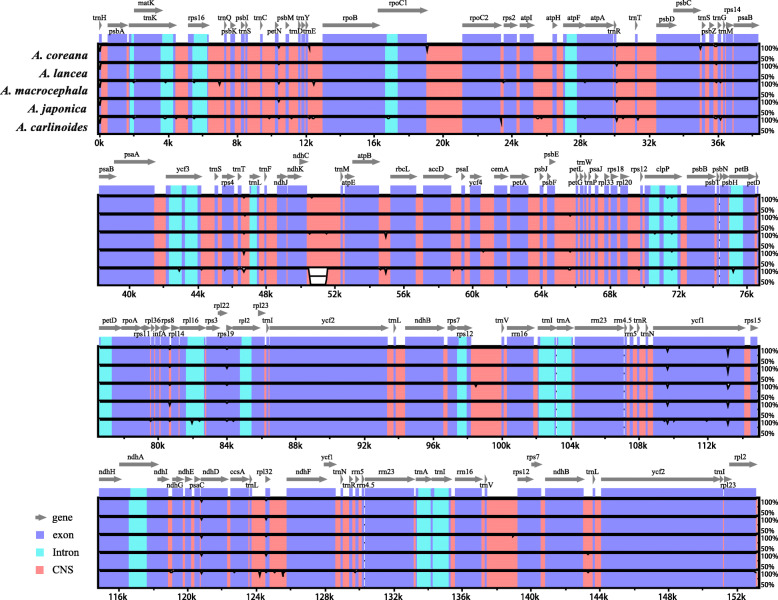
Visualization of genome alignment of the chloroplast genomes of six *Atractylodes* species using *A. chinensis* as a reference by mVISTA. The x-axis represents the coordinate in the chloroplast genome. The sequence similarity of the aligned regions is shown as horizontal bars indicating the average percent identity within 50–100%

A comparative analysis based on mVISTA was performed in the six chloroplast genomes of *Atractylodes* to determine the level of divergence (Fig. 5). The results indicate high sequences similarities across the chloroplast genome suggesting that the chloroplast genomes are highly conserved. The IR regions and the coding regions are more conserved than the single copy regions and the noncoding regions. The coding regions of the *clpP*, *ycf1* and *rps19* genes are more variable than the coding regions of other genes.

Additionally, we compared single nucleotide substitutions and nucleotide diversity in the total, LSC, SSC and IR regions of the chloroplast genomes (Table 3). Six *Atractylodes* chloroplast genomes were aligned with a matrix of 153,560 bp with 445 variable sites (0.29%) and 31 parsimony-informative sites (0.02%). The average nucleotide diversity value was 0.001. The IR regions have the lowest nucleotide diversity (0.0003) and the SSC regions have the highest diversity (0.0018).

**Table 3:** Variable site analyses of Atractylodes chloroplast genomes

Regions	Length	Variable sites		information sites		Nucleotide diversity
		Numbers	%	Numbers	%	
LSC	84,501	310	0.3669	22	0.0260	0.0013
IR	25,153	19	0.0755	1	0.0040	0.0003
SSC	18,753	97	0.5173	7	0.0373	0.0018
Complete chloroplast genome	153,560	445	0.2898	31	0.0202	0.0010

**Fig. 6:**
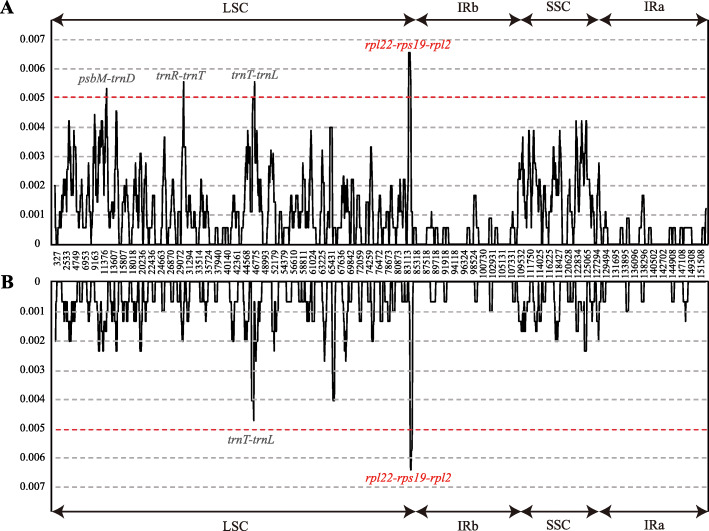
Sliding-window analysis of the whole chloroplast genomes. (A) All six *Atractylodes* species (B) Five species used in herbal medicine excluding *A. carlinoides.* Window length: 600 bp; step size: 100 bp. X-axis: position of the midpoint of a window. Y-axis: nucleotide diversity of each window

The nucleotide diversity was measured by DNAsp to identify the mutation hotspot regions in the whole *Atractylodes* chloroplast genomes (Fig. 6). Nucleotide diversity values within 600 bp vary from 0 to 0.00656 in group A and from 0 to 0.00633 in group B. The region *rpl22-rps19-rpl2* has the highest Pi values (Pi = 0.00656) followed by the other three spacer regions (Pi > 0.005) including *psbM-trnD*, *trnR-trnT*^(GGU)^, and *trnT*^*(UGU)*^-*trnL* in the group A dataset; all these features are located in the LSC region. On the other hand, group B shares lower diversity; however, the region *rpl22*-*rps19*-*rpl2* still has the highest diversity. The variability of four identified mutation hotspot regions was tested together with three universal chloroplast DNA barcodes (*matK*, *rbcL* and *trnH-psbA*). The universal DNA barcodes had lower variability than that of the newly identified markers.

### Phylogenetic analysis

**Fig. 7:**
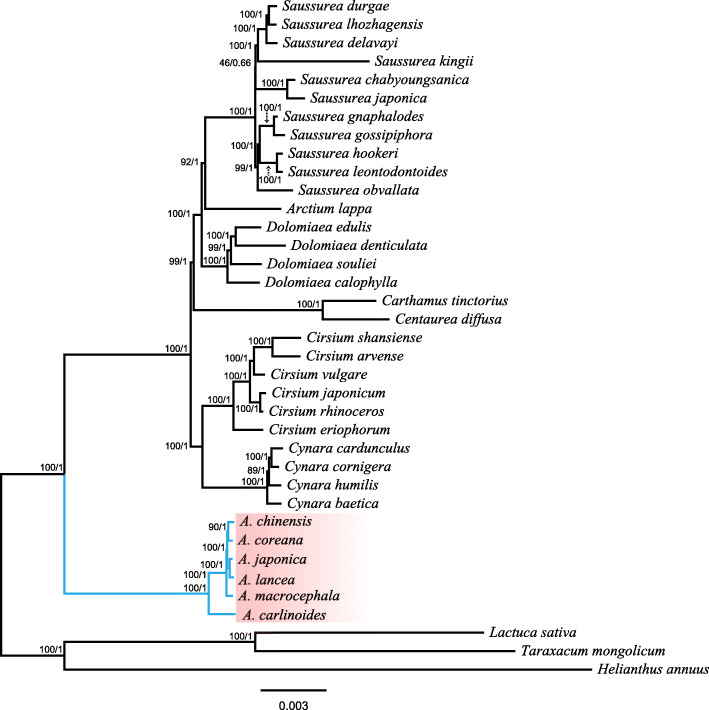
Phylogenetic tree constructed using the maximum likelihood (ML) and Bayesian inference (BI) methods based on the whole chloroplast genomes from 37 different species. The numbers above the branches represent the ML bootstrap values/BI posterior probabilities

Using the whole plastome sequences, we preformed phylogenetic analysis of the 37 tribe Cynareae species. The topologies of the ML and BI trees are essentially consistent (Fig. 7). *Atractylodes* is a sister of other Cynareae species and *Atractylodes* species form a monophyletic group with 100% support. Within *Atractylodes*, *A. carlinoides* is located at the base. *A. japonica* and *A. lancea* cluster into a subclade and form a sister relationship with the subclade of *A. chinensis* and *A. coreana*. The phylogenetic relationship carried out by indels is consistent with the results obtained by using the whole plastome sequences (Fig. S1).

## Discussion

### The chloroplast genome of *Atractylodes*

In this study, the chloroplast genomes of six *Atractylodes* species were sequenced by the NGS methods. The chloroplast genome size ranges from 152,294 bp (*A. carlinoides*) to 153,261 bp (*A. macrocephala*). All species have 113 genes, including 79 protein-coding genes, 30 transfer RNA genes and four ribosomal RNA genes, in the chloroplast genome. In this study, we did not annotate the *ycf15* and *ycf68* genes because we identified them as pseudogenes containing several internal stop codons [18]. In certain cases, *ycf2*, *rpl23* and *accD* are absent from the chloroplast genomes [19–21]; however, but these genes are indeed present in *Atractylodes.* The chloroplast genome is conserved similar to the majority of plants; no rearrangement events were detected in all species. The mVISTA results and nucleotide diversity tests indicate high similarities between the chloroplast genomes implying that the divergence of the *Atractylodes* chloroplast genome is lower than that of other species [6, 22, 23].

We identified 114 indels in the *Atractylodes* chloroplast genome, including 30 SSR-related and 84 non-SSR-related. Indels are another important class of genetic variation in addition to nucleotide substitutions. In SSR-related indels, polymerase slippage results in addition or deletion of short spans of sequences that repeat at one side of the region flanking the indels [24]. The majority of the SSR-related indels are primarily detected in the AT-regions [25]. Intramolecular recombination and hairpins or the stem-loop secondary structure are causing the majority of the non-SSR-related mutations [26]. In most cases, the non-SSR-related indels are more frequent than SSR-related indels [26]. In *Atractylodes*, the non-SSR-related indels are more than two-fold frequent than the SSR-related indels. Nucleotide divergence is significantly correlated with size and abundance of the nearby indels [27–29], which indicate that indels are associated mutation hotspots.

### Phylogenetic relationships

*Atractylodes* is a small genus with six species. However, due to low genetic divergence and similar morphology, the systematic relationship of *Atractylodes* remains unclear. Use of several chloroplast markers, such as (*atpB-rbcL, psbB-F, trnL-F*), for phylogenetic resolution is insufficient to draw the firm conclusions about the interspecies relationships in *Atractylodes* [15–17]. Therefore, sampling of additional more genetic features is expected to improve phylogenetic resolution. Large-scale application of high-throughput technology enhanced availability of the sequencing of the whole chloroplast genomes resulting in resolution of closely related species using plastome sequences [5, 30, 31].

In this study, we used the plastome sequences to assess the phylogenetic relationships within *Atractylodes*. The results indicate the presence of the deep phylogenetic relationships in *Atractylodes*. *A. carlinoides* is located at the base of the genus and *A. macrocephala* was separated later [16]. The taxonomic controversy of *Atractylodes* is predominantly concentrated in the *A. lancea* complex, which includes four species *A. coreana, A. chinensis*, *A. japonica* and *A. lancea. A. japonica* is distributed in Northeast China, Korean, and Japan, and it has a synonym of *Atractylodes lancea* in “Flora of China”. According to the chloroplast genome data, *A. japonica* and *A. lancea* are clustered into a clade. The morphology of *A. japonica* differs from the other species of the *A. lancea* complex; for example, the leaves of *A. japonica* have long petioles and are generally divided or completely divided into 3–5 lobes [16]. *A. chinensis* is considered a species or a variant of *A. lancea* var. *chinensis* or a synonym of *A. lancea*; this classification has been an issue of controversy. Based on the morphology, *A. chinensis* is difficult to distinguish from *A. lancea.* Phylogeny of *Atractylodes* indicates that *A. chinensis* is a sister of *A. coreana* (Fig. 7). *A. lancea* is a polytype species based on the morphology [32, 33] and ITS and trn*L*-*F* of multiple individuals [16]. *A. coreana* is distributed only in the Liaodong and Shadong Peninsulas. Peng et al. treated this species as a synonym of *A. chinensis* based on the *trnL-F* and ITS data*.* In this study, the chloroplast genome data provide effective markers to infer the phylogeny of *Atractylodes.* However, sampling of additional individuals of the species of the *A. lancea* complex can provide additional evidence of evolutionary history.

### Potential highly variable chloroplast barcodes

Increasing number of case studies indicate that the universal DNA barcodes have lower divergence and poor discriminatory power [12]. In *Atractylodes*, these regions lack variability and may lead to unsuccessful identification and confusing relationships between the species (Table 4). *Atractylodes* is an important commodity of Chinese medicinal plants; the lack of genomic resources for *Atractylodes* is the main obstacle to taxonomy, genetics, identification and conservation. Chloroplast genome sequences provide an opportunity to illustrate the genome evolution and generate valuable genetic resources for further studies. The mutation events in the chloroplast genome are not universally randomly distributed within the sequence and are concentrated in certain regions forming the “hotspot” regions [12]. Comparison of the chloroplast genome sequences is an effective strategy to identify the mutation hotspots and these highly variable regions can be used as the specific DNA barcodes. In this study, we identified four hypervariable regions, including *rpl22-rps19-rpl2*, *psbM-trnD*, *trnR-trnT*^*(GGU)*^, and *trnT*^*(UGU)*^*-trnL*.

**Table 4:** The variability of the hypervariable markers and universal chloroplast DNA barcodes

Markers	Length	Variable sites		information sites		Nucleotide Diversity
		Numbers	%	Numbers	%	
*rbcL*	1434	3	0.21	0	0.00	0.0007
*matK*	1520	9	0.59	1	0.07	0.0021
*trnH-psbA*	393	3	0.76	1	0.25	0.0031
*rbcL + matK + trnH-psbA*	3347	15	0.45	2	0.06	0.0016
*psbM-trnD*	827	10	1.21	1	0.12	0.0053
*trnR-trnT*	715	10	1.40	0	0.00	0.0056
*trnT-trnL*	921	10	1.09	0	0.00	0.0050
*rpl22-rps19-rpl2*	1105	13	1.18	3	0.27	0.0066
*psbM-trnD + trnR-trnT + trnT-trnL + rpl22-rps19-rpl2*	3568	43	1.21	4	0.11	0.0042

The *psbM-trnD* region is a part of the *trnC-trnD* intergenic marker which is divided into three intergenic regions, *trnC-petN*, *petN-psbM*, and *psbM-trnD*. The *psbM-trnD* region has a long history of use in the plant phylogenetic studies [34]. The *trnT*^*(UGU)*^*-trnL* was a part of *rps4-trnT*^*(UGU*^ and was suggested by [35] as a high level variability marker; the region is used in certain groups for molecular studies of low taxonomic markers [36]. The *rpl22-rps19-rpl2* region consists of two intergenic spaces (*rpl22-rps19* and *rps19-rpl2*) and a coding gene (*rps19*) with an average size of 1104 bp; this region is the most variable marker in the *Atractylodes* chloroplast genome (Fig. 6 and Table 4). However, this marker was not extensively used in plant phylogeny and DNA barcoding. The *trnR-trnT*^*(GGU)*^ was identified for the first time in this study and can be used in the subsequent studies.

## Conclusions

In this study, we sequenced and assembled the complete chloroplast genomes of six *Atractylodes* species, providing valuable genomic resource of this genus. Based on whole chloroplast phylogenomic analysis, the relationship within the genus was clearly resolved for the first time. Meanwhile, the comparative analysis of chloroplast genomes generated variable regions which could be used as the specific DNA barcodes. All the obtained genetic resources will facilitate future studies in population genetics, species identification and conservation biology of *Atractylodes*.

## Methods

### Plant materials and DNA extraction

The materials of *Atractylodes* species were obtained from the herbarium of PE (Herbarium, Institute of Botany, CAS) and CMMI (Institute of Chinese Materia Medica, China Academy of Chinese Medical Sciences). Total DNA was extracted following the method of Li et al. [37] and purified by a Wizard DNA cleanup system (Promega, Madison, WI, USA). DNA quality was assessed by spectrophotometry and the integrity was evaluated using a 1% (w/v) agarose gel.

### Sequencing, assembly, and annotation

Total DNA was fragmented to 350 bp fragments by ultrasound. A paired-end library was constructed by a NEBNext UltraTM DNA library prep kit, and PE150 sequencing was performed on the Illumina HiSeq X Ten platform.

NGS QC toolkit was used for quality control and to filter the low quality reads. Contigs were assembled from the high quality paired-end reads by using the SPAdes 3.6.1 program [38] (Kmer = 95). Then, the chloroplast genome contigs were selected by the Blast program using the chloroplast genome of *A. chinensis* (NC037484) as a reference [39]. Subsequently, the selected contigs were assembled using Sequencher 4.10. Geneious 8.1 [40] was used to map all reads to the assembled chloroplast genome sequence to verify the assembling accuracy. The complete chloroplast genome sequences were annotated with Plann [41] using *A. chinensis* (NC037484) as a reference, and a ring diagram was created by using OrganellarGenomeDRAW [42].

### Analysis of microstructural mutation events

Six chloroplast genomes were aligned using MAFFT V7 software [43], and manually adjusted using Se-al 2.0 [44]. The variable mutation sites and parsimony information sites in the chloroplast genome were assigned using MEGA 7.0 [45].

Simple sequence repeats (SSR) were predicted using the Genome-wide Microsatellite Analyzing Tool Package (GMATA) software [46] with the search parameters set at > 10 repeat units for mononucleotide, > 5 repeat units for dinucleotide, > 4 repeat units for trinucleotide, and > 3 repeat units for tetranucleotide, pentanucleotide, and hexanucleotide SSRs.

Based on the aligned sequence matrix, the indels were manually validated and divided into two categories, including SSR-related and non-SSR-related (normal indels). *A. chinensis was* used as a reference to determine the size and position of the indels events.

### Comparison of the chloroplast genomes and divergent hotspot identification

Comparison of the whole chloroplast genomes of *Atractylodes* was performed by the mVISTA program (http://genome.lbl.gov/vista/mvista/submit.shtml) with the Shuffle-LAGAN mode. Sequence of *A. chinensis* was used as a reference. The nucleotide diversity of the chloroplast genome was calculated based on the sliding window analysis using the DnaSP v5.10 software [47]. The window length was set to 600 bp with a 100 bp step size. *A. carlinoides* has a well distinguished morphology and five other species were used as a traditional Chinese medicine. Two data sets were created for this analysis: (1) all six species data set (group A) and (2) five medical species (group B).

### Phylogenetic reconstruction

Thirty-seven chloroplast genome sequences were used for phylogenetic analysis, including six *Atractylodes* samples and 31 samples of other species of *Cynareae* and *Lactuceae* from the GenBank (Table S2). All chloroplast genome sequences were aligned using MAFFT and ambiguous alignment regions were trimmed by Gblocks 0.91b [48].

Phylogenetic analysis was carried out using the maximum likelihood (ML) and Bayesian inference (BI) methods. The optimal model TVM + F + I + G4 was calculated by Modelfinder based on the BIC standard (recommended by the software) [49]. ML calculations were performed using the IQ-tree [50], and the sampling was repeated 1000 times. Bayesian inference (BI) of the phylogenies was implemented with MrBayes [51]. The Markov chain Monte Carlo (MCMC) analysis was run for 10,000,000 generations. The trees were sampled every 1000 generations and the initial 25% were discarded as burn-in. Finally, average standard deviation of the split frequencies <0.01 was verified. And the phylogenetic analysis by using obtained indel data (including SSRs) was conducted by MEGA 7.0 in ML method.

## Supplementary Information

**Additional file 1 **Fig. S1. Phylogenetic tree constructed using the maximum likelihood (ML) methods based on the obtained indel data of *Atractylodes* species.

**Additional file 2.** Table S1. Detailed information of indels.

**Additional file 3.** Table S2. Information on the chloroplast genome downloaded from Genbank for phylogenetic analysis.

## Data Availability

Six annotated chloroplast sequences have been submitted to NCBI (https://www.ncbi.nlm.nih.gov) with accession numbers: MT834519 ~ MT834524. The reference sequence for assembly and annotation was obtained from NCBI with accession number: NC037484, (https://www.ncbi.nlm.nih.gov/nuccore/NC_037484), Information for phylogenetic analysis download from Genbank can be found in Table S2. All raw reads are available in the short sequence archive under accession no. PRJNA692669.

## Abbreviations

BI: Bayesian inference
IR: inverted repeat region
LSC: large single copy region
ML: Maximum Likelihood
rRNA: Ribosomal RNA
SSR: simple sequence repeats
SSC: small single copy region
tRNA: Transfer RNA
